# Personalized intravenous regional anesthesia

**DOI:** 10.4103/1658-354X.65117

**Published:** 2010

**Authors:** Pamela Flood

**Affiliations:** *Associate Professor of Clinical Anesthesiology, Columbia University, New York, USA E-mail pdf3@columbia.edu*

Intravenous regional anesthesia is simple, low risk anesthetic for surgery of distal extremity. Drawbacks to its use include tourniquet pain, slow onset of action and rapid resolution of postoperative analgesia. Non-steroidal anti-inflammatory drugs and clonidine have previously been shown to improve postoperative analgesia and clonidine improves tourniquet pain.[1] In this issue of *Saudi Journal of Anaesthesia*, Elmetwaly and coauthors have added two additional effective adjuvant drugs to our armamentarium for intravenous regional anesthesia.[2]

In a randomized, double blind study of 75 patients having hand surgery, 25 received lidocaine supplemented with ketamine 0.1 mg/kg, another 25 received lidocaine supplemented with 200 g nitroglycerine and the other 25 received lidocaine alone. Both the adjuvant treatments were associated with more rapid onset of sensory and motor block after initiation of anesthesia and slower resolution. Less tourniquet pain was reported by patients who received adjuvant treatment with a larger effect size in the ketamine group. Both the treatments reduced postoperative pain.

Metabolism of nitroglycerine to NO within the vasculature causes vasodilation within the blocked area which would be expected to speed the onset of the block. Intrathecal NO donors are thought to reduce central sensitization and treatment with systemic nitroglycerine has been shown to prolong the effect of intrathecal sufentanil and may act synergistically with intrathecal neostigmine. However, since nitroglycerine is evanescent in the vasculature, the mechanism by which it would reduce postoperative pain after 45 minutes of metabolism within the vasculature during surgery is less clear. There is no known peripheral affect.

The effect of ketamine on postoperative pain is easier to understand. Ketamine is a well-known analgesic drug that acts at least in part as an N-methyl-D-aspartate acid (NMDA) antagonist. Ketamine may enhance intra-operative conditions including tourniquet pain by blocking NMDA receptors expressed on peripheral c-fibers in the forearm. It is metabolized in the liver by N-demethylation to nor-ketamine; so, it would be expected to be released from the arm unchanged after deflation of the tourniquet. A significant postoperative pain effect from ketamine 0.1 mg/kg is not surprising.

Regardless of the mechanism of action, practitioners of intravenous regional anesthesia now have four reasonable adjuvant agents from which to choose. Individual patient characteristics should guide the choice of adjuvant drug. Patients with poor peripheral vasculature may particularly benefit from the vasodilatory action of nitroglycerine added to the local anesthetic for peripheral regional anesthesia. Patients with severe ongoing pain or opioid tolerance would be expected to have additional benefit from adjuvant clonidine or ketamine as these drugs are known to be efficacious in these conditions. Non-steroidal analgesics are efficacious as adjuvant analgesics in many postoperative settings but care should be taken when bone healing and renal perfusion are an issue.

In summary, Elmetwaly *et al*. have described two potentially useful adjuvant drugs for intravenous regional anesthesia. A clear delineation of which drug is best for which class of patient will have to wait for future clinical trials but the availability of additional options is undoubtedly better.

## References

[1] Choyce A, Peng P (2002). A systematic review of adjuncts for intravenous regional anesthesia for surgical procedures. Can J Anaesth.

[2] Elmetwaly KF, Hegazy NA, Aboelseoud AA, Alshaer AA (2010). Does the use of ketamine or nitroglycerin as an adjuvant to lidocaine improve the quality of intravenous regional anesthesia?. Saudi J Anaesth.

